# Synthetic Medical Document Generation Pipeline (MedDocSynth) for Optical Character Recognition: Development and Validation Study on Korean Clinical Documents

**DOI:** 10.2196/91313

**Published:** 2026-10-21

**Authors:** Mikel Lainsa, Javier Aguirre, Moon Hyeyoon, Won Yejun, Woojin Jang, Jong Eun Park, Won Chul Cha

**Affiliations:** 1 Department of Digital Health, Samsung Advanced Institute for Health Sciences and Technology (SAIHST), Sungkyunkwan University, Seoul, Republic of Korea; 2 Samsung Medical Center, Seoul, Republic of Korea; 3 Department of Emergency Medicine, Sungkyunkwan University School of Medicine, Samsung Medical Center, 81 Irwon-ro, Gangnam-gu, Seoul, 06351, Republic of Korea, 82 2-3410-2053

**Keywords:** optical character recognition, document synthesis, Korean medical documents, data generation pipeline, healthcare AI

## Abstract

**Background:**

The development of structured optical character recognition (OCR) systems for Korean medical documents is hindered by strict privacy regulations that limit access to real clinical data. Existing synthetic document frameworks are primarily designed for generic layouts and Western languages, and fail to capture the complex formatting, bilingual terminology, and hierarchical field semantics required for clinical OCR and document-understanding tasks in hospital environments.

**Objective:**

This study aimed to develop and validate a privacy-preserving, template-driven pipeline for generating realistic synthetic Korean medical documents with structured XML annotations, enabling OCR and document-understanding model training in settings where access to real clinical data is restricted.

**Methods:**

We designed MedDocSynth, a 7-stage synthetic document generation pipeline that reconstructs authentic medical document layouts from publicly available references, incorporates clinician-guided template selection, and uses a structured placeholder system encoding 17 medical data types. Human-curated templates were populated with exemplar content and tagged using a delimiter-based scheme, after which GPT-4o-mini generated diverse synthetic values for each field. The pipeline produced 1000 fully synthetic DOCX documents with corresponding XML annotations encoding hierarchical structure, title-answer relationships, and datatype metadata. We evaluated text fidelity, lexical diversity (type-token ratio, measure of textual lexical diversity [MTLD], n-grams, and Pareto), and compared the system with existing synthetic document pipelines. A Qwen2.5-VL model was fine-tuned exclusively on the synthetic dataset and evaluated on 50 real-world Korean clinical documents to assess OCR accuracy, information extraction, and table reconstruction.

**Results:**

The pipeline achieved 100% placeholder coverage, 99.68% format correctness, and 91.56% semantic accuracy. Lexical diversity analysis demonstrated high variability in free-text fields (MTLD≈456) and expected repetitiveness in structured datatypes. Compared with existing pipelines (SynthText, SynthTiger, and DocSynth), the proposed system uniquely supports complex clinical forms, bilingual content, and structured XML annotations. When trained solely on the synthetic data, Qwen2.5-VL achieved the highest real-world performance, with a character accuracy of 86.01%, word accuracy of 78.25%, field accuracy of 67.18%, datatype accuracy of 64.14%, and table reconstruction tree-edit-distance similarity score of 71.09%. Models without fine-tuning performed substantially worse, including GPT-5-mini.

**Conclusions:**

We present the first synthetic data generation pipeline specifically tailored to Korean medical documents, enabling the creation of realistic, richly annotated datasets for structured OCR without exposing patient information. The synthetic data significantly improves OCR performance on real clinical documents, demonstrating the pipeline’s potential utility for hospital deployment and its potential extensibility to other languages and health care systems.

## Introduction

### Background

Optical character recognition (OCR) systems play a central role in digitizing hospital documents by converting scanned images into machine-readable text, enabling downstream structured data extraction and database integration. However, their development is constrained by strict privacy regulations that restrict access to real patient records [1], particularly in languages such as Korean, where institutional formats, complex layouts, and writing conventions differ markedly from those in English-language datasets. As a result, existing clinical OCR systems often exhibit limited generalizability and weak performance in real-world hospital environments [2].

In hospitals, paper-based medical records are routinely scanned and archived as image files within electronic medical record (EMR) systems. While this approach preserves document content, the images remain nonsearchable and nonextractable. When clinicians need to access specific information―particularly in time-critical settings, such as emergency departments―they must manually locate and transcribe data from scanned images, a process that is both inefficient and error-prone. Structured OCR systems, capable of automatically extracting and organizing text from diverse Korean medical documents, would significantly improve clinical workflow efficiency and patient safety.

Structured OCR addresses these limitations by automatically recognizing content and structure from scanned medical documents and organizing them into XML-annotated, EMR-compatible formats. This approach enables (1) automated extraction of structured clinical fields (patient demographics, medication lists, allergy records, and laboratory results) from individual documents; (2) EMR-compatible output that preserves field-level semantics (title-answer relationships and datatypes) for direct database integration; (3) elimination of manual transcription, reducing errors, and clinician cognitive load [3,4]; and (4) a foundation for downstream applications, such as cross-document search and longitudinal patient data aggregation once integrated into hospital information systems.

Synthetic data generation provides a promising solution by enabling the creation of realistic training corpora without exposing sensitive information [5]. However, existing frameworks―including SynthText [6], SynthTiger [7], and DocSynth (DS) [8]―are designed for generic document structures and Western languages, while Korean OCR benchmarks, such as AI Hub OCR [9], CLOVA OCR [10], and KAIST Scene Text [11], lack the hierarchical layout modeling, semantic field pairing, and domain-specific medical terminology required for clinical documents. Crucially, this limitation is not unique to Korean; no published pipeline for Japan, Taiwan, or any other non-English health care system addresses the combined challenge of full-page clinical form image synthesis, intradocument bilingual content, and structured field-level annotation for OCR training [12-14]. Recent layout analysis datasets [15-17] rely on weak supervision from digital sources and fail to capture the irregular formatting, mixed-language text, and scanning artifacts typical of hospital records.

These limitations motivate a specialized, privacy-preserving pipeline capable of generating realistic Korean medical documents for clinical OCR and document-understanding tasks. To our knowledge, no equivalent pipeline exists for non-English clinical environments. Existing non-English work falls into two distinct categories, neither of which meets this need: (1) synthetic clinical text generation for natural language processing tasks (such as named entity recognition or deidentification), which produces no document images, no form layouts, and no field-level annotations [12,13,18-20]; and (2) generic multilingual document image synthesis pipelines, which cover multiple languages but apply no domain-specific clinical content, no bilingual medical form templates, and no datatype-aware semantic annotation [21,22]. This gap is particularly acute in health care systems where clinical documents routinely mix 2 languages within a single form—a challenge documented for Korean and, notably, for Taiwanese medical records, yet unaddressed by any existing document synthesis pipeline. We address this gap by developing MedDocSynth, a modular, template-driven synthetic document generation pipeline that integrates curated document structures with AI-assisted text generation. Datatype-aware placeholders ensure consistent, medically plausible bilingual (Korean-English) content, while paired XML annotations capture title-answer relationships and fine-grained structural metadata, enabling OCR and document-understanding model training without exposing real clinical data.

### Objectives

This study aims to develop a privacy-conscious, template-driven pipeline for synthesizing realistic Korean medical documents with structured XML annotations (title-answer and datatype) across diverse document classes. We further aim to quantify textual realism and variability using lexical diversity metrics (type-token ratio [TTR], measure of textual lexical diversity [MTLD], n-grams, and Pareto) and establish baseline comparisons against existing synthetic pipelines. Importantly, we validate the utility of the pipeline by evaluating OCR and document understanding performance on real clinical documents, comparing a fine-tuned Qwen 2.5 VL model against both its GPT-5-mini and Qwen 2.5 VL baseline model, to demonstrate that the generated data are usable in real-world scenarios.

### Main Contribution

Our key contributions are:

- We introduce the first synthetic data generation pipeline tailored specifically for Korean medical documents, capturing realistic clinical layouts, bilingual content, and domain-specific terminology.
- We design a template-driven system capable of producing full-page documents with complex structured elements, including tables, form fields, multicolumn layouts, and standardized clinical formatting.
- We propose a new XML-based annotation schema, inspired by DocTags [23], that encodes hierarchical document structure, datatype metadata, and semantic title-answer relationships for downstream OCR and document understanding tasks.
- We provide a scalable, extensible framework that supports diverse clinical document types and enables privacy-preserving, reproducible training, and benchmarking of OCR models in health care settings.
- We validate the pipeline using real clinical documents, demonstrating that models fine-tuned on the synthetic data (Qwen 2.5 VL) outperform non–fine-tuned and baseline language models such as GPT-5-mini, confirming the practical utility of our approach.

## Methods

### Overview

To address the scarcity of publicly available Korean medical document datasets and privacy constraints, we developed a synthetic data generation pipeline to create realistic, privacy-safe OCR training corpora. The pipeline reconstructs representative document templates and populates them with AI-generated, medically plausible bilingual (Korean-English) content. Each generated document is paired with a structured XML annotation capturing hierarchical title-answer relationships and semantic datatypes.

### MedDocSynth: Synthetic Document Generation Pipeline

Figure 1 illustrates the end-to-end workflow used to construct our synthetic Korean medical document dataset. MedDocSynth consists of 7 sequential stages, beginning with template sourcing and expert selection and ending with the production of fully populated documents and their corresponding structured annotations. This approach allows the creation of complex layout synthetic document generation. Each of the following sections is aligned with each of the steps referenced in Figure 1.

**Figure 1.**
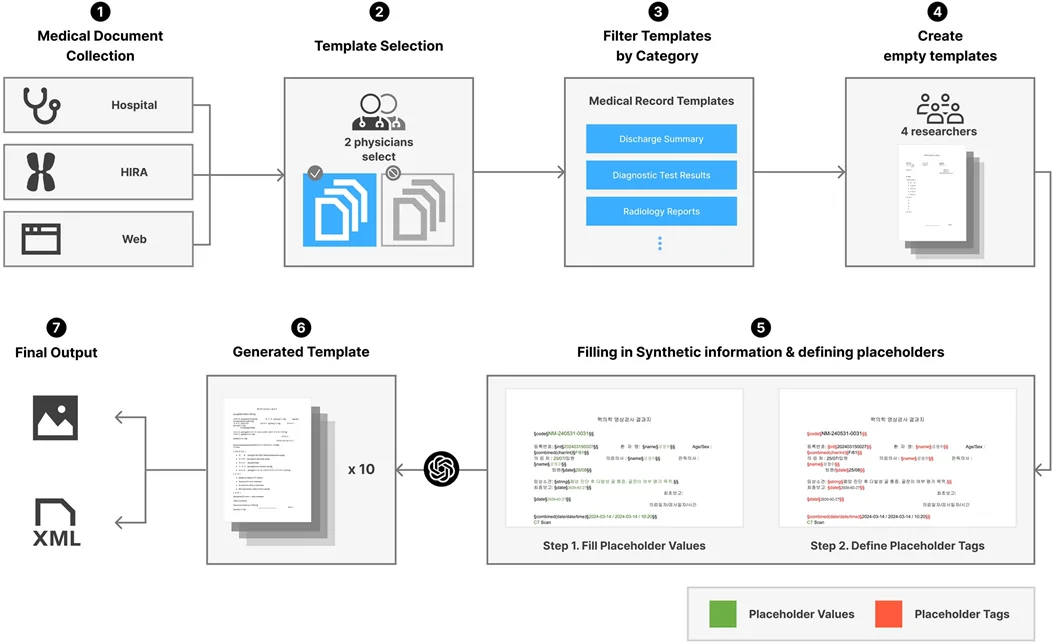
Overview of the MedDocSynth pipeline for synthetic dataset generation, divided into 7 steps. Step 1: medical documents are collected from three sources (hospital, Health Insurance Review and Assessment Service, and web-based repositories). Step 2: two physicians review and filter the documents based on relevance, structural differences, and other quality criteria. Step 3: the selected documents are categorized to guide template creation. Step 4: using the filtered documents, empty templates are constructed to mimic the structure and layout of the real documents. Step 5: in a two-stage process, these templates are filled with human-generated placeholder content, after which tags are assigned to each content element. Step 6: using these tags and placeholders, GPT-4o-mini generates synthetic data by replacing the tagged elements, producing 10 synthetic records per template. Step 7: finally, document images and corresponding XML files are exported to form the final dataset.

### Medical Document Collection

The step 1 of the pipeline as seen in Figure 1 begins with the compilation of real-world medical document samples that serve mainly as structural references. We aggregated publicly accessible materials from the following three sources: (1) hospital-issued forms and publicly released examples, (2) Health Insurance Review and Assessment Service (HIRA) documents, and (3) medical document samples available on official or educational websites. No patient information or protected health data were included; mainly the layout, structure, and formatting style of these documents were used. These references form the foundation from which empty, fully synthetic templates are later manually constructed by human labelers.

### Template Selection by Clinical Experts

To ensure that the dataset covers the most clinically representative and structurally diverse document types, 2 licensed physicians conducted an expert-driven template selection process. They reviewed all collected document structures and identified those considered high-priority for clinical relevance, frequency of use, and structural diversity. Only templates approved by both clinicians were retained, ensuring that downstream synthetic documents reflect real hospital documentation practices while remaining fully privacy-preserving.

### Filtering Templates by Category

The curated set of candidate documents was next organized into medical record categories based on expert opinion of 2 clinicians. Categorization serves the following two purposes: (1) ensuring balanced coverage across distinct document classes, and (2) enabling class-specific generation strategies for documents with unique structural characteristics (eg, narrative-heavy summaries vs table-dominated test results). This stage produces a structured taxonomy that guides all subsequent template construction and synthetic data generation.

We identified 12 core document classes that represent the most clinically relevant and structurally diverse types of hospital records—discharge summaries, diagnostic test results, medical imaging results, pathology results, surgical records, emergency records, consultation records, inpatient initial assessment records, physician’s orders, transfer records, discharge transfer records, and medication records.

### Creation of Empty Document Templates

A team of 4 researchers then reconstructed each selected document type as an empty DOCX template, replicating only the layout, formatting, section structure, and tabular arrangements from the reference examples. All content fields were left blank to avoid any leakage of real patient information. These templates capture intraclass variability by incorporating natural differences in formatting, table organization, headings, and field arrangements. The output of this stage is a high-quality library of layout-accurate but fully deidentified 100 medical document templates, which will serve as the seed of the synthetic document generation. This step provides us with the capability of tailor-making a highly complex structure dataset for any desired scenario.

### Defining Placeholders and Filling Synthetic Information

#### Overview

To enable automated content generation, each empty template was populated with a structured placeholder system. We adopted the section symbol (§) as the delimiter, as it does not conflict with typical medical characters. Each placeholder follows the syntax §<datatype>§<example_value>§§, where the first and second § delimit the datatype identifier (eg, name, date, and code) and the second § and closing §§ delimit the representative example value. For instance, §name§Kim Jiyoon§§ encodes a name-type field with “Kim Jiyoon” as the exemplar. The system supports 17 datatypes covering the diversity of medical records; full definitions and examples are provided in Table S2 in Multimedia Appendix 1. The templates were processed in 2 steps—placeholder value generation and placeholder tag definition.

#### Placeholder Value Generation

As addressed in Figure 1 step 5, first, a team of four trained labelers manually filled every template instance with realistic synthetic example text following the datatype constraints. This process produced 100 fully populated, human-curated template documents containing both natural linguistic variation and correct field-level structure.

#### Placeholder Tag Definition

Second, the same labelers then inserted placeholder tags around each example value using the delimiter notation described above, thereby specifying the datatype and semantic role anticipated for automated generation.

Among the 17 datatypes, the select type requires distinct handling because it represents categorical choices rather than free-text generation. The system first identifies selection markers in the placeholder text—including circle (○/●) and square (□/■) styles―extracts each option regardless of its initial state, randomly selects between 1 and 3 options, and reassigns the selected/unselected symbols accordingly. For example: Input: ◦ Diabetes • Hypertension ◦ None

Output: • Diabetes ◦ Hypertension • None

For placeholders without visual markers, delimiter-separated options (eg, M/F or Yes|No) are parsed and one option is randomly selected. Unlike other datatypes, select placeholders are processed locally without API calls.

Together, these steps produce a set of 100 labeled, example-filled templates that serve as ground truth specifications for the generation stage. In the subsequent phase of the pipeline, each template is automatically instantiated ten times, expanding the initial 100 human-created documents into 1000 fully synthetic, consistently annotated records.

### Generation of Synthetic Documents

#### Overview

With all templates and placeholders defined, the pipeline automatically generates the final synthetic documents. This stage comprises 2 parallel processes—content generation, which populates each placeholder with new synthetic values, and annotation generation, which produces a structured XML file encoding the document’s semantic and structural metadata.

#### Content Generation

For each template, the GPT-4o-mini model replaces every placeholder with newly generated synthetic content, adhering to the datatype, structure, and formatting constraints encoded during the template creation stage. Each placeholder is processed via an independent API call with temperature=0.7 to balance diversity and plausibility, and max_tokens=10,000 to accommodate long free-text fields such as radiology findings, surgical descriptions, and clinical progress notes, which may span multiple sentences or paragraphs.

During content generation, the pipeline processes DOCX documents by iterating through the document body in sequential order, distinguishing between paragraph elements and table elements. In Korean medical documents, single-column tables are frequently used as layout containers rather than data structures—for instance, to frame narrative sections or group related content within bordered regions. The pipeline detects single-column tables and processes their cell contents as regular text elements rather than tabular structures, preventing downstream models from misinterpreting layout-only tables as data tables.

#### Annotation Generation

Each generated document instance is paired with a structured XML annotation file. The annotation format uses a simplified XML schema inspired by DocTags [23], with 2 primary elements: <text> for textual content and <table> for structured tabular data, subdivided into <row>, <hcel> (header cell), and <ncel> (normal cell).

Content originating from placeholder substitution receives class=“answer” along with a datatype attribute specifying the field type, while all non-placeholder text―such as field labels, section headings, and static form content―is automatically assigned class=“title.” For example:

<text class=“title”>Patient Name</text>

<text class=“answer” datatype=“name”>Park Minjae</text>

For combined datatypes (eg, entries such as “58/M”), an additional subtype attribute records the constituent types:

<text class=“answer” datatype=“combined” subtype=“int/gender”>58/M</text>

Tabular content follows a hierarchical<table>→<row>→ cell structure. For merged cells, the span attribute records the number of columns spanned. An example annotation for a diagnostic test result table is:

<table>

<row>

  <hcel class=“title”>Test Name</hcel>

  <hcel class=“title”>Result</hcel>

  <hcel class=“title”>Reference</hcel>

 </row>

<row>

  <ncel class=“title”>Hemoglobin</ncel>

  <ncel class=“answer” datatype=“float”>13.2</ncel>

  <ncel class=“answer” datatype=“range”>

   12.0‐-16.0</ncel>

 </row>

</table>

The full XML schema with tag definitions and attribute descriptions is provided in Table S1 in Multimedia Appendix 1.

### Final Output

#### Overview

This process yields 1000 fully synthetic documents, each consisting of (1) a DOCX file containing realistic synthetic medical content and (2) a parallel XML annotation encoding hierarchical structure, title-answer relationships, and datatype metadata. Examples of generated documents with their corresponding templates and XML annotations can be seen in Figure S1 in Multimedia Appendix 2. Together with the placeholder definitions in Table S2 in Multimedia Appendix 1, these materials provide sufficient detail to reproduce the core pipeline.

#### Synthetic Data Evaluation

##### Overview

The goal of our evaluation is to determine whether the proposed pipeline can generate synthetic documents that are realistic, diverse, and semantically reliable. To this end, we conducted 3 complementary experiments assessing textual correctness, linguistic variability, and overall pipeline performance.

We conducted the following three experiments to assess the pipeline: (1) text fidelity, (2) lexical diversity, and (3) pipeline comparison.

##### Text Fidelity

The objective of this experiment is to determine whether the pipeline produces realistic and semantically reliable placeholders. To evaluate the quality of synthetically generated documents, we adopt a placeholder-level evaluation strategy rather than holistic document-level scoring [24]. Each generated placeholder was evaluated for presence, format correctness, and semantic accuracy. Validation included checking datatype constraints (eg, numeric ranges and date formats), verifying structured dependencies such as combined fields, and allowing naturalistic variation including multiple date styles and flexible spacing patterns. All placeholders across synthetic documents were first reviewed solely by a licensed nurse, who served as the primary labeler. Subsequently, the nurse (labeler), a biomedical specialist, and 2 computer science engineers jointly re-examined the placeholders―the nurse and biomedical specialist for semantic accuracy and medical realism, and the engineers for datatype constraint validation

##### Lexical Diversity

To measure linguistic variability in the generated documents, we computed TTR and MTLD. TTR reflects the proportion of unique tokens relative to the total number of tokens, formally defined as “TTR”=V/N where V is the vocabulary size and N is the total token count; a higher TTR indicates greater lexical richness, although it is known to decrease as document length grows [25]. MTLD addresses this length sensitivity by computing the mean length of sequential token runs. We also analyzed n-gram frequency distributions to capture repetition at the level of multitoken sequences, and applied Pareto analysis to identify the proportion of n-gram types accounting for 80% of total token mass―a low type coverage at this threshold indicates high repetition and low generative diversity [26].

##### Pipeline Comparison

We compared our system with SynthText, SynthDoc, SynthTiger, and DS. The comparison focused on layout coverage, the use of domain-specific terminology, handling of bilingual content, and the quality of structured annotation produced by each system. The aim is to demonstrate that our pipeline supports document types and linguistic structures that are not adequately covered by existing approaches.

### Real-World Validation (Model Training and Fine-Tuning)

We fine-tuned the Qwen2.5-VL visual language model (VLM) on our synthetic dataset using a train-in-synthetic, test-on-real (TSTR) paradigm with 1000 synthetically generated samples. The model simultaneously predicts (1) recognized text, (2) title and answer classification, (3) semantic datatypes, and (4) table structures.

Training details include:

- Precision: bf16

- Hardware: 8× NVIDIA A100 80 GB GPUs
- Epochs: 4
- Learning rate: 1×10^−5^
- Batch size: 1 (gradient accumulation 16)
- Parallelization: DeepSpeed ZeRO Stage 3

Post training, the model was quantized using activation-aware weight quantization (AWQ) to create Qwen2.5-VLFinetuned-AWQ for efficient inference.

### Evaluation Dataset and Metrics

An independent real-world test set of 50 clinical document images spanning 11 document categories was constructed to evaluate generalization under realistic conditions. The dataset includes documents originating from 10 tertiary hospitals in South Korea, and more than 25 documents, between primary and secondary hospitals capturing substantial interinstitutional variation in document formats and styles.

Unlike curated benchmark datasets, the images reflect real-world acquisition conditions. The dataset includes both scanned documents and photographs captured using mobile devices, exhibiting diverse artifacts such as shadows, perspective distortion, motion blur, compression noise, partial occlusion, and document wear (eg, folds and faded text).

We intentionally prioritize diversity and realism over scale, as variability in clinical document acquisition conditions is a primary challenge for deployment. This heterogeneity introduces significant visual and structural variability, making the dataset a challenging and realistic test bed for clinical document understanding systems.

Test set samples differ from the training samples in several important ways. The training data consists of clean captures of synthetically generated documents, without blur or additional augmentation. In contrast, the test set contains documents affected by a wide range of real-world noise and artifacts. Only 3 documents in the test set originate from the same institution as the templates used to generate the training data. However, even in these limited cases, the documents remain distinct and independent; all remaining documents come from previously unseen institutions. While the layouts belong to the same general families defined during data generation, they are structurally and textually distinct from those observed in the training set.

Although limited to 50 documents, the dataset contains a large number of structured elements (fields, key-value pairs, and table cells), enabling fine-grained evaluation beyond document-level accuracy. In total, the evaluation covers 2377 structured elements (1011 text fields and 1366 annotated values) across all documents. The test set was constructed independently from the template generation process, and no exact document layouts or images were reused during synthetic data creation. Given the limited number of documents, results should be interpreted with consideration of dataset size, although the large number of structured evaluation elements provides additional robustness. Detailed statistics are provided in Table S3 in Multimedia Appendix 1.

### Annotation Protocol and Interannotator Agreement

Ground truth annotations were produced by 4 annotators with complementary expertise (1 clinical nurse, 1 biomedical specialist, and 2 engineers), ensuring both clinical validity and technical consistency. Annotation was performed at the level of individual XML lines, where each unit corresponds to a structured element (eg, text blocks, table cells, or rows). All 50 documents were annotated to establish the reference standard.

To assess annotation reliability, a representative subset of documents was independently annotated by multiple annotators, covering diverse layouts such as free-text entries, tables, and mixed-format clinical forms. This subset comprised 1274 annotated units used for agreement analysis.

Interannotator agreement was measured using Cohen κ at the line level. Prior to computation, annotations were normalized to reduce the impact of XML alignment discrepancies (eg, line shifts due to missing or merged elements), which would otherwise artificially penalize agreement. Minor variations, such as spacing or small spelling differences were retained.

The overall Cohen κ was 0.73, indicating substantial agreement. Lower agreement was primarily observed in structurally complex cases (eg, nested tables or ambiguous layouts), where disagreements were typically due to representational differences rather than true semantic inconsistencies.

All disagreements were resolved through consensus-based adjudication, where annotators jointly reviewed conflicting cases and agreed on a final annotation. These adjudicated labels were used as the definitive ground truth for all reported evaluations.

Metrics include:

- OCR accuracy: character error rate (CER) and word error rate (WER), measuring transcription quality at the character and word levels.
- Information extraction: field accuracy, datatype accuracy, and *F*_1_-scores (macro, weighted, and per-datatype), evaluating how well the model identifies each field, classifies its semantic type, and extracts its corresponding value.
- Table structure: tree-edit-distance similarity (TEDS), assessing structural correctness of predicted tables, including row and cell organization and alignment of text to its intended cell.

Comparisons were made between: Qwen2.5-VL-Finetuned, Qwen2.5-VL-Finetuned-AWQ, Base Qwen2.5-VL, GPT-5Mini (zero-shot), GPT-5-Mini (3-shot), and GPT-5-Mini (5-shot).

To provide a fair comparison, we include both zero-shot and few-shot prompting baselines for GPT-5-Mini. In the few-shot setting, 3-shot and 5-shot prompts were constructed by providing annotated examples from the training set; prompt examples have been added in Table S6 in Multimedia Appendix 1.

To assess the statistical robustness of our results given the limited test set size, we performed bootstrap resampling over the 50 test documents. For each metric, 5000 resamples with replacement were drawn at the document level, and 95% CIs were computed as the 2.5th and 97.5th percentiles of the resulting distribution. Aggregation followed the same pooling logic as the primary evaluation, corpus-level pooling for CER and WER, and element-level pooling for field and datatype accuracy, ensuring that bootstrap means exactly reproduce the observed values. All reported results include these intervals.

### Ethical Considerations

This study was reviewed by the Institutional Review Board of Samsung Medical Center and granted a waiver of full ethics review under expedited procedures (IRB case SMC 2026-05-119). All synthetic documents were generated from publicly available structural references and contain no patient information. Real-world clinical document images used for validation were fully deidentified prior to use. No patient-identifiable data was collected, accessed, or stored at any stage of this study. This study complies with *Journal of Medical Internet Research* (*JMIR*) policy and Committee on Publication Ethics (COPE) guidelines regarding ethics reporting.

## Results

### Text Fidelity

#### Overview

We evaluated text fidelity to assess how accurately each placeholder was instantiated according to its intended datatype and exemplar format. For every placeholder occurrence, we measured 3 aspects—presence consistency, format correctness, and semantic accuracy. Table 1 summarizes results across all datatypes.

**Table 1.** Text fidelity evaluation across datatypes. The table is separated in upper part being clinical information datatype and lower part other datatypes making reference to lexical datatypes useful for information extraction. The table describes high accuracy in both format correctness with a 99.68% correctness and semantic accuracy with 91.56%.

Datatype	Total occurrences, n	Format correctness, n (%)	Semantic accuracy, n (%)
Name	291	290 (99.66)	291 (100)
Date	331	329 (99.4)	331 (100)
Time	145	145 (100)	145 (100)
Phone number	20	20 (100)	20 (100)
Gender	13	13 (100)	13 (100)
Address	12	12 (100)	12 (100)
Code	102	102 (100)	92 (90.2)
Amount	69	69 (100)	69 (100)
Unit	20	20 (100)	13 (65)
Other string	840	839 (99.88)	684 (81.43)
Other int	210	210 (100)	210 (100)
Other float	205	205 (100)	205 (100)
Arrow	45	45 (100)	43 (95.56)
Symbol	25	25 (100)	4 (16)
Range	14	14 (100)	14 (100)
Combined	33	33 (100)	32 (96.97)
Select	112	108 (96.43)	99 (88.39)
Total	2487	2479 (99.68)	2277 (91.56)

The system achieved 100% presence consistency, indicating no placeholders were omitted. Format correctness remained high at 99.68%, confirming the generator effectively replicated expected syntactic patterns. Semantic accuracy averaged 91.56%, demonstrating strong contextual reliability while highlighting areas for refinement.

Detailed inspection revealed 4 main failure patterns. The observed errors can be further categorized according to their origin, which falls into two primary sources: (1) VLM’s inherent limitations and (2) suboptimal synthetic training data.

#### Field Label–Answer Misalignment

It occurred primarily in string fields, where content belonged to the wrong information category. This is attributable to the synthetic training data, in which all field values were represented under a generic “string” type without semantic distinction, allowing heterogeneous content to be assigned to any field indiscriminately.

#### Entity Plausibility Failures

It includes structurally correct but fictitious entities, such as nonexistent medications (“sky medicine”) or implausible departments (“Department of Neuroscience”). These are predominantly attributable to VLM hallucination, arising when sufficient grounding information is absent rather than from noise in the training corpus.

#### Logical Consistency Violations

It includes impossible dates or unit–value mismatches (eg, temperature recorded as “500 mg”). These are attributed to suboptimal synthetic training data. Critically, table structures in the synthetic data often had ambiguous spatial layouts between adjacent cells. This caused a systematic table-offset error, where the model misidentified the target cell for a given value. This is a known limitation of the cell-localization algorithm rather than a failure of the pipeline’s semantic understanding; the extracted value itself was semantically valid, but was mapped to an adjacent field due to layout ambiguity.

#### Selection Incompleteness

This includes occasional failures in choosing among multiple options (eg, checkboxes or categorical values), mainly in symbol, arrow, or combined types. These errors arose from both VLM limitations ― where selection markers (eg, filled circles) were misrecognized as visually similar characters ― and inconsistent rendering of selection elements in synthetic documents.

Overall, the pipeline maintains high syntactic precision and strong semantic reliability. The most clinically salient errors, such as unit-field mismatches, stem from table spatial ambiguity in training data rather than semantic misinterpretation, and are addressed by the guardrails described in the Discussion section.

### Lexical Diversity and Patterns

To characterize linguistic variability across datatypes, we analyzed TTR, the MTLD, and representative *n*-gram patterns. Table 2 summarizes results for both single-word and multiword fields.

Across multiword fields, the string datatype demonstrated the greatest lexical richness, with an MTLD of approximately 456 and a moderate TTR (0.22). This reflects sustained vocabulary variety and the broad semantic space typical of free-text clinical entries. In contrast, other multiword datatypes such as address and combined fields showed substantially lower

MTLD values (3-28) despite comparable or higher TTR values, indicating that their multitoken sequences are structurally repetitive rather than lexically diverse.

Patterns in single-word fields further highlight differences in constraint. Highly variable identifiers—notably code (TTR=0.70) and phone number (TTR=0.66)—exhibited strong diversity due to large pools of unique alphanumeric or numeric values. More formatted fields, including float, time, and amount, showed markedly lower diversity (TTR<0.15), consistent with tightly constrained token spaces. The lowest variation occurred in gender (TTR=0.075), reflecting an inherently small categorical vocabulary.

**Table 2.** TTR^a^ and MTLD^b^ values show that free-text fields (eg, other string) exhibit substantially higher lexical variability, while structured or categorical fields display lower diversity due to their formulaic structure―although some categorical datatypes with larger value spaces (eg, code and phone number) remain comparatively more varied than highly constrained ones such as gender or combined.

Datatype	Samples, n	TTR	MTLD
Single-word fields			
Name	1690	0.2399	—^c^
Date	2050	0.1579	—
Time	350	0.1229	—
Phone number	170	0.6647	—
Gender	80	0.0750	—
Code	680	0.6986	—
Amount	670	0.1444	—
Unit	200	0.4000	—
Other Int	1400	0.2238	—
Other Float	1890	0.1384	—
Multiword fields			
Other string	4930	0.2187	456.1820
Address	80	0.3129	28.1390
Combined (gender/int)	70	0.1800	7.1795
Combined (int/gender)	10	0.2000	3.3333
Combined (name/string)	10	0.4359 1	10.1250

a TTR: type-token ratio.

b MTLD: measure of textual lexical diversity.

c Not applicable.

### N-Grams and Pareto Analysis

As presented in Table 3, the *n*-gram distribution patterns corroborate these trends. Free-text fields (other strings) produced diverse bigrams and trigrams due to their low formulaic nature and showing some moderate occurrence encompassing medical routines and dosage expressions (eg, “once daily” and “ibuprofen mg”) suggesting their common usage, therefore, showcasing high MTLD and broad linguistic coverage. In contrast, structured fields such as address displayed highly repetitive, template-driven geographic sequences (eg, “Seoul City, Gangnam-gu”), consistent with narrow semantic roles and their comparatively low MTLD. The original table in Korean is added in Table S4 in Multimedia Appendix 1.

Pareto frequency curves exhibit similar distinctions; free-text fields showed flatter accumulation profiles as seen in Figure 2B plot blue curve, indicating less dominance by a small set of high-frequency tokens, while structured or categorical fields exhibited steep curves characteristic of repetitive patterns. Combined fields, with limited sample size and predefined compositional templates, showed the strongest frequency concentration and correspondingly low MTLD values.

Overall, the lexical diversity, *n*-gram, and Pareto analyses provide a coherent picture—open-text fields maintain substantial variability and semantic breadth, whereas numeric, categorical, and structured fields are intentionally constrained, yielding predictable and repetitive patterns. These differences reflect meaningful datatype-specific characteristics rather than artifacts of the synthetic data generation process showcasing realistic data generation diversity.

**Table 3.** Top bigrams and trigrams by datatype. Free-text entries show broad n-gram variety but also a small number of moderately frequent, clinically relevant phrases (eg, “once daily”―78 occurrences out of 4930), which is expected and mirrors real-world clinical usage; these repeated patterns contribute to high measure of textual lexical diversity without indicating excessive templating. In contrast, structured fields such as addresses produce template-like geographic sequences (eg, repeated “gangnam-gu” tokens), reflecting both narrow semantic roles and a dataset-specific geographic bias.

Datatype and top bigrams (2 grams)	Top trigrams (3 grams)
String (4930 samples)	
“once daily” (n=78) “per day” (n=65) “q h” (every h; n=64)	“mg if needed” (n=33) “U A EDTA” (n=30) “mg per day” (n=30)
“ibuprofen mg” (n=63)	“every evening dose” (n=30)
“in progress” (n=59)	“once before meal” (n=30)
Address (80 samples)	
“Seoul City, Gangnam-gu” (n=21) “Gangnam-gu, Teheran-ro” (n=19) “Building/Room” (n=13)	“Seoul, Gangnam-gu, Teheran-ro” (n=10) “Seoul City, Gangnam-gu, Teheran-ro” (n=9) “Seoul City, Gangnam-gu, Samsung-ro” (n=7)
“Seoul, Gangnam-gu” (n=11)	“Seoul, Songpa-gu, Jamsil-dong” (n=6)
“Busan, Haeundae-gu” (n=9)	“Busan Metropolitan City, Haeundae-gu, Jung-dong” (n=3)
Combined name+string (10 samples)	
“Dermatologist specialist” (n=3) “Younghee Lee, Dermatologist” (n=2) “Psychology professor” (n=2)	“Younghee Lee, Dermatologist specialist” (n=2) “Younghee Lee, Pediatrician” (n=2) “Minsu Lee, Psychology professor” (n=1)
“Younghee Lee, Pediatrician” (n=2)	“Minjae Lee, Psychology professor” (n=1)
“Pediatrician” (n=2)	“Minji Lee, Psychology researcher” (n=1)

**Figure 2.**
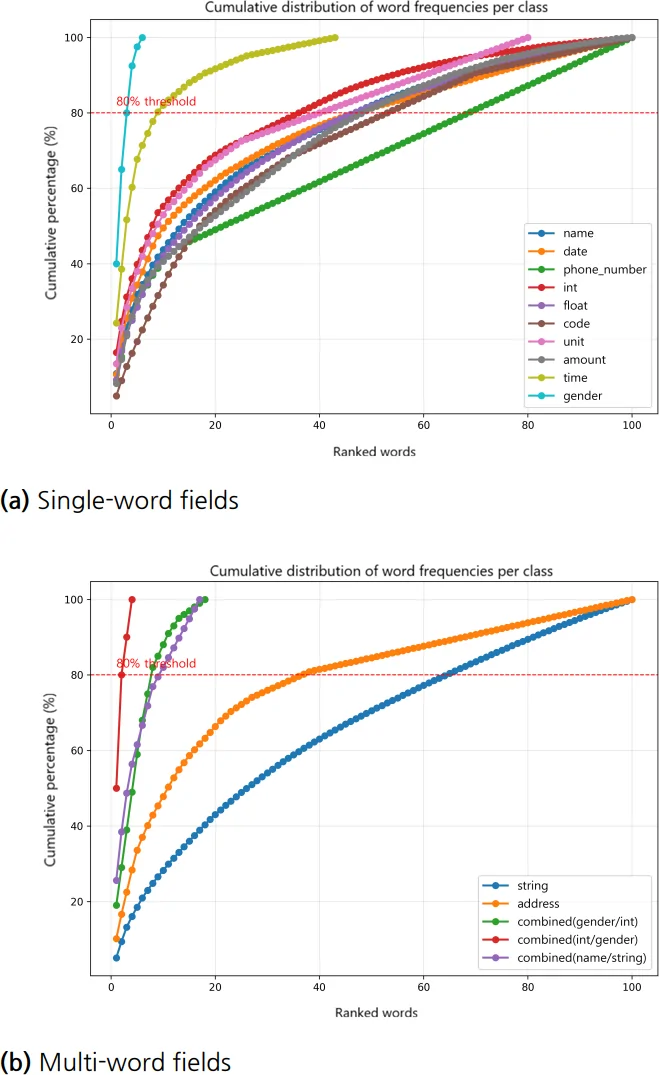
(A) Single-word fields show steep curves for structured categories (eg, gender, unit, and code) and flatter curves for more variable fields such as names and dates. (B) Multi-word fields reveal strong contrasts: free-text fields (string) exhibit the flattest, most diverse profiles, while structured fields (address) and combined fields display steep, highly repetitive distributions. These patterns align with the n-gram and measure of textual lexical diversity analyses, reflecting the inherent variability of open-text fields vs the constrained nature of structured datatypes.

### Comparison With Other Data Generation Pipelines

Table 4 displays a comparison among different pipelines. Data generation pipelines for document and text synthesis vary widely in their design objectives and capabilities. Early pipelines such as SynthText (ST), TextRenderer (TR), TextRecognitionDataGenerator (TRDG), and SynthTiger (STi) focus primarily on text line image generation. These systems are effective at producing high-quality text in multiple fonts, languages, and formats, but their outputs are limited to simple text lines. They cannot generate complex document structures, handle tables or forms, or incorporate domain-specific content, making them unsuitable for applications such as medical document generation.

Pipelines such as DocCreator (DC) and DS extend this functionality to full-page document synthesis. DC generates documents by extracting layout and style information from a given template, while DS allows the creation of academic paper–style documents following user-defined layouts. While these approaches enable some structured document generation, they are restricted in scope; DC is mainly limited to paragraph-based layouts, and DS focuses on academic formatting. Neither system adequately supports the complex forms, tables, headers, footers, or domain-specific content typical of medical documents.

Our pipeline MedDocSynth addresses these limitations by providing template-driven generation of Korean medical documents, supporting diverse document types and layouts. It enables the creation of structured content including complex tables, forms, headers, and footers, while incorporating domain-specific medical terminology. This combination of layout flexibility, structured content, and domain relevance allows MedDocSynth to generate realistic synthetic medical documents suitable for training and evaluating OCR and document understanding systems.

The comparison in Table 4 highlights the differences between existing pipelines and our approach. It shows that while SynthText, TextRenderer, TextRecognitionDataGenerator, and SynthTiger excel at basic text rendering, and DC and DS allow limited structured document generation, only MedDocSynth supports comprehensive structured document synthesis with domain-specific content, multiple layouts, and complex forms and tables.

**Table 4.** Comparison of synthetic document generation pipelines. Earlier systems (SynthText, TextRenderer, TextRecognitionDataGenerator, and SynthTiger) primarily support text-line rendering and lack structured document capabilities, while DocCreator and DocSynth provide limited page-level layout synthesis without domain adaptation. In contrast, our pipeline MedDocSynth uniquely supports template-driven Korean medical documents with complex forms, tables, multilayout structures, AI-generated clinical content, and fully structured XML annotations―enabling end-to-end generation of realistic medical documents for OCR^a^ and document understanding research.

Feature or capability	SynthText	TextRenderer	TextRecognitionDataGenerator	SynthTiger	DocCreator	DocSynth	MedDocSynth
Basic text rendering	✓^b^	✓	✓	✓	✓	✓	✓
Basic paragraph rendering	NS^c^	NS	NS	✓	NS	✓	✓
Multiple text format support	✓	✓	✓	✓	✓	✓	✓
Korean / Non-latin script support	Limited	✓	✓	✓	✓	✓	✓
Structured document generation	NS	NS	NS	✓	NS	✓	✓
Multiple layout generation	NS	NS	NS	✓	NS	✓	✓
Complex table generation	NS	NS	NS	NS	NS	✓	✓
Complex form generation	NS	NS	NS	NS	NS	NS	✓
Domain-specific (medical) content generation	NS	NS	NS	NS	NS	NS	✓
Template driven generation	NS	NS	NS	✓	NS	✓	✓
Structured annotations	—^d^	—	—	—	—	—	✓

a OCR: optical character recognition.

b Given tool supports the function.

c NS: given tool does not support the function.

d Not applicable.

### Real-World Model Evaluation

#### Overview

We evaluated 4 models on 50 real-world document images across 11 categories—Qwen2.5-VL-Finetuned, its quantized variant AWQ, the base nonfinetuned Qwen2.5-VL, zero-shot GPT-5-Mini, 3-shot GPT-5-Mini, and 5-shot GPT-5-Mini. Metrics included OCR accuracy (CER or WER), field and datatype classification, and table reconstruction (TEDS).

#### Overall Performance

As described in Table 5, the fine-tuned model achieved the highest performance, that is, character accuracy 86.01% (CER=13.99%), word accuracy 78.25% (WER=21.75%), field accuracy 67.18%, and datatype accuracy 64.14%.

In comparison, GPT-5-Mini in the zero-shot setting lagged significantly (word accuracy 65.23% and field accuracy 43.95%). Introducing few-shot prompting improved performance across most metrics; 3-shot prompting increased word accuracy to 67.07% and field accuracy to 46.36%, while 5-shot prompting showed similar performance (word accuracy 66.86% and field accuracy 46.3%). These results indicate that in-context examples help mitigate schema unfamiliarity and improve structured extraction.

However, even with few-shot prompting, GPT-5-Mini remained substantially below the fine-tuned model across all evaluation dimensions, particularly in structured outputs, such as datatype prediction and table reconstruction. Improvements from 3-shot to 5-shot prompting were limited, suggesting diminishing returns from additional in-context examples.

The AWQ variant showed moderate degradation (WER 26.8% and datatype accuracy 51.72%), demonstrating deployable efficiency with minimal loss. To evaluate this efficiency, a speed benchmark was conducted on a DGX Sparks using Qwen-2.5-VL-Finetuned and Qwen-2.5-VL-Finetuned-AWQ. Inference was performed on all 50 images in the test set, and the mean inference time and SD (in seconds) were recorded. The baseline model achieved 123.5 (SD 79.6) seconds, whereas the AWQ variant achieved 53.4 (SD 34.4) seconds.

The nonoverlapping CIs across all primary metrics confirm that performance differences between the fine-tuned model and all baselines are statistically robust despite the limited test set size.

**Table 5.** Comprehensive evaluation of OCR^a^ quality, information extraction accuracy, and table reconstruction fidelity. Metrics include character or word error rates (CER^b^ or WER^c^), character- and word-level recognition accuracy, field and datatype prediction accuracy, and table structure alignment measured via TEDS^d^. Each value reports the observed mean followed by the 95% bootstrap confidence interval in parentheses, estimated via 5000 resamples over the 50-document test set. Bold denotes the best result per metric.

Model	OCR accuracy				Information extraction accuracy		Table accuracy
	CER (%) ↓mean (95% CI)	WER (%) ↓mean (95% CI)	r accuracy (%) ↑mean (95% CI)	Word accuracy (%) ↑mean (95% CI)	Field accuracy (%) ↑mean (95% CI)	Datatype accuracy (%) ↑mean (95% CI)	TEDS average (%) ↑mean (95% CI)
Qwen2.5-VL-Finetuned	13.99 (9.84-18.76)	21.75 (16.21-27.94)	86.01 (81.24-90.16)	78.25 (72.06-83.79)	67.18 (59.77-74.77)	64.14 (55.16-72.07)	71.09 (57.16-85.10)
Qwen2.5-VL-Finetuned-AWQ	17.26 (11.89-23.56)	26.80 (21.04-33.06)	82.74 (76.44-88.11)	73.20 (66.94-78.96)	56.89 (48.79-65.25)	51.72 (43.26-60.44)	55.68 (37.84-75.13)
GPT-5-Mini	19.92 (15.47-24.62)	34.77 (29.33-40.44)	80.08 (75.38-85.53)	65.23 (59.56-70.97)	43.95 (38.83-49.55)	40.34 (32.86-47.80)	18.13 (13.20-29.10)
GPT-5-Mini-3-shot	19.27 (14.21-24.85)	32.93 (26.70-39.29)	80.73 (75.15-86.79)	67.07 (60.71-73.30)	46.36 (39.29-53.67)	47.81 (38.82-55.70)	42.71 (25.90-60.99)
GPT-5-Mini-5-shot	18.49 (13.75-23.58)	33.14 (26.96-39.65)	81.51 (76.42-86.25)	66.86 (60.35-73.04)	46.30 (39.47-53.03)	44.93 (37.12-52.15)	41.28 (22.66-61.27)
Qwen2.5-VL	73.70 (66.88-80.02)	82.15 (76.11-88.48)	26.30 (20.32-33.25)	17.85 (12.56-24.76)	39.41 (32.11-47.51)	31.38 (24.22-38.13)	3.98 (1.12-8.74)

a OCR: optical character recognition.

b CER: character error rate.

c WER: word error rate.

d TEDS: tree-edit-distance similarity.

#### Field and Datatype Classification

The fine-tuned model as presented in Table 6 achieved macro-*F*_1_=0.8020 and weighted-*F*_1_=0.8051 for answer-title classification, outperforming all baselines.

Few-shot prompting improved GPT-5-Mini performance over the zero-shot setting (macro-*F*_1_ increasing from 0.6172 to 0.6423 for 3-shot), but remained well below the fine-tuned model. Gains were primarily observed in title classification, while answer extraction recall remained limited, indicating challenges in consistently identifying structured content fields.

**Table 6.** Precision, recall, and *F*_1_-score for classifying metadata (title) and content (answer) fields extracted by our optical character recognition system. Titles correspond to metadata labels defined in the XML ground truth, while answers represent the content associated with those labels. Accurate classification of these fields is essential for enabling structured information retrieval.

Model	Answer			Title			Macro-*F*_1_-score	Weighted-*F*_1_-score
	Precision	Recall	*F*_1_-score	Precision	Recall	*F*_1_-score		
Qwen2.5-VL-Finetuned	0.859	0.7892	0.8226	0.7164	0.8595	0.7815	0.802	0.8051
Qwen2.5-VL-Finetuned-AWQ	0.8416	0.6574	0.7382	0.6227	0.8309	0.7119	0.725	0.727
GPT-5-Mini	0.7719	0.451	0.5693	0.8653	0.5401	0.665	0.6172	0.61
GPT-5-Mini-3-shot	0.8	0.4383	0.5663	0.8885	0.6028	0.7183	0.6423	0.6305
GPT-5-Mini-5-shot	0.7978	0.4288	0.5578	0.8818	0.617	0.726	0.6419	0.6288
Qwen2.5-VL	0.7849	0.4729	0.5902	0.6727	0.4411	0.5329	0.5615	0.5658

Per-datatype performance for the fine-tuned model was highest for structured fields (date 0.8493, phone 0.8333, code 0.8095, and address 0.8000) and lowest for rare or ambiguous types (amount and gender). Quantized AWQ performance remained competitive for some low-frequency fields.

Additionally, datatype extraction accuracy can be seen in Table S5 in Multimedia Appendix 1.

#### Table Structure Reconstruction

For TEDS, as presented in Table 5, the fine-tuned model achieved an average of 71.09%, with a maximum TEDS of 100%, indicating perfect table reconstruction for at least 1 complex example.

GPT-5-Mini performed poorly in the zero-shot setting (18.13%), but improved substantially with few-shot prompting (42.71% for 3-shot and 41.28% for 5-shot). This highlights that providing structural examples helps the model better approximate table layouts. However, performance remained significantly below the fine-tuned model, indicating persistent difficulty in capturing precise hierarchical and spatial relationships.

The base Qwen2.5-VL model performed poorly (3.98%), highlighting the importance of domain-specific adaptation.

Overall, results suggest that while few-shot prompting improves performance, it remains challenging to achieve high accuracy on schema-based, layout-intensive document understanding tasks without task-specific fine-tuning.

Additionally, to better characterize table reconstruction performance, we conducted an analysis of TEDS scores across document types and error modes. The proposed model achieved an average TEDS score of 71.7%, with multiple instances of perfect structural reconstruction (TEDS=1.0), indicating strong performance on well-structured tables. Class-wise analysis revealed substantial variability depending on document complexity; near-perfect performance was observed in outpatient, surgical, and pulmonary reports (TEDS≥0.93), while lower performance was observed in diagnostic and medical imaging documents (TEDS=0.31 and 0.54, respectively), which typically contain more irregular layouts.

Error analysis showed that the predominant failure mode was row mismatch, where the model over- or undersegmented table rows, particularly in dense or structurally complex tables. In contrast, column mismatches and general structural misalignment were less frequent. Notably, identical scores between structure-only and full TEDS indicate that errors are primarily structural rather than related to content recognition or text alignment. Additionally, a subset of documents did not contain explicit tabular structures and were therefore excluded from TEDS evaluation, highlighting the dependency of this metric on the presence of detectable tables. Representative failure cases are provided to illustrate these challenges.

### Summary of Results

Across all metrics, fine-tuning on synthetic data significantly improved OCR and document understanding. Largest gains were observed in structured information extraction and table reconstruction (improvements of 13‐55 percentage points). The AWQ variant confirms efficient inference is feasible without major performance loss. These results validate the effectiveness of our synthetic Korean medical document pipeline for training VLMs for its potential for real-world scenarios. Additionally, training on clean (noise-free) synthetic data proved effective for generalization to noisy real-world inputs. While the underlying cause cannot be definitively established, we hypothesize that it was already Qwen-2.5-VL’s native capability, which likely enables it to handle noise and visual variability even when fine-tuned on clean data.

## Discussion

### Principal Findings

This study presents the first comprehensive synthetic data generation pipeline specifically designed for Korean medical documents, addressing a critical gap in OCR research for clinical applications. Rather than targeting immediate clinical deployment, this work addresses a foundational prerequisite, that is, enabling the development of structured OCR systems in settings where strict privacy regulations have historically made real clinical data inaccessible for training. Our template-driven approach successfully generates 1000 diverse medical documents across 12 distinct clinical document classes, demonstrating both the feasibility and effectiveness of AI-powered synthetic data generation in the medical domain.

The lexical diversity analysis reveals important insights about the nature of medical document content, with free-text fields (strings) exhibiting exceptionally high lexical diversity (MTLD>450 for strings), while structured fields demonstrate expected patterns of repetition that accurately reflect standardized medical formatting conventions.

MedDocSynth introduces several methodological innovations that distinguish it from existing synthetic data generation approaches. The placeholder-based system using section symbols (§) as delimiters provides robust content generation control, while AI-powered generation using GPT-4o-mini produces medically plausible content reflecting authentic clinical terminology and mixed-language patterns. The XML-based annotation format, inspired by DocTags but simplified for medical documents, provides rich metadata enabling downstream models to understand semantic roles and context. The comparative analysis reveals our approach’s unique positioning, while traditional pipelines like SynthText excel at basic text rendering, and advanced systems like DC support some structured generation, only MedDocSynth combines template-driven Korean medical layouts, AI-filled clinical content, and structured XML output for comprehensive medical document synthesis.

While the pipeline comprises multiple components—including template design, placeholder-based content generation, and structured XML annotation—not all contribute equally to downstream performance. Based on our observations during development, the most critical factor is the supervision signal defined by the labeling guidelines and XML schema. In particular, consistent annotation of title-answer relationships and datatype labels proved essential, as small inconsistencies in labeling led to substantial variability in model behavior. In contrast, factors such as visual realism (eg, noise or blur) and exact layout fidelity had a comparatively smaller impact, as the model demonstrated strong robustness to diverse document structures. The placeholder-based system and synthetic content generation played a complementary role by reinforcing semantic consistency, but were secondary to the clarity and consistency of the annotation framework. These findings suggest that the primary contribution of our pipeline lies in its structured, datatype-aware supervision design rather than in photorealistic document synthesis.

An additional methodological strength arises from our separation of model versions during development and validation. Synthetic data were generated using GPT-4o-mini, while evaluation leveraged GPT-5-Mini, ensuring that observed performance gains are not artifacts of model memorization or self-consistency bias. This design provides a conservative yet realistic assessment of how synthetic documents support downstream OCR and document-understanding tasks, highlighting that strong results stem from the quality of the template-driven generation rather than the intrinsic knowledge of any single LLM.

The clinical relevance of this work extends beyond technical OCR performance by addressing critical challenges in Korean health care digitization, where strict data protection laws have historically impeded effective OCR solution development. By providing a privacy-compliant, on-premise deployable dataset covering diverse document types from discharge summaries to laboratory reports, our work enables hospitals to develop OCR systems without compromising patient confidentiality. The practical utility demonstrated here through real-world validation represents a necessary first step toward clinical integration, with deployment studies, clinician evaluations, and workflow assessments constituting important and clearly identified directions for future work. This template-driven approach offers a generalizable framework that could be adapted for other languages and medical systems, contributing to global health care digitization efforts while maintaining the clinical realism essential for robust OCR model training.

From a governance and ethical perspective, even when using fully synthetic medical data, responsible use remains essential. Risks related to potential reidentification, unintended memorization, and scope creep must be carefully managed through clear usage boundaries, institutional oversight, and adherence to applicable privacy regulations [27].

Beyond data governance, safe deployment of clinical document understanding systems requires explicit safeguards against downstream extraction errors. To this end, we propose a layered postprocessing guardrail framework integrated downstream of the model. This includes (1) rule-based schema validation to enforce datatype and format constraints, (2) controlled vocabulary matching for closed-domain entities (eg, medications and specimen types), (3) cross-field logical consistency checks to detect violations across related fields (eg, temporal inconsistencies), and (4) human-in-the-loop review for ambiguous or high-risk cases. Such guardrails are particularly important in mitigating silent failure modes (eg, structurally valid but semantically incorrect outputs) and provide a practical pathway toward safe real-world deployment.

### Limitations

This study has some limitations that should be considered when interpreting the results. First, we adopted a TSTR evaluation strategy to assess whether fully synthetic medical documents can support OCR and document-understanding model training under strict privacy constraints. This choice reflects realistic deployment conditions in which access to real clinical data is severely limited. While alternative training strategies—such as hybrid synthetic-real setups or iterative retraining schemes—may further improve performance, the TSTR configuration used in this study is sufficient to demonstrate the feasibility and practical utility of the proposed approach.

Second, the evaluation was conducted using a vision-language model with integrated OCR capabilities rather than a standalone OCR engine. As a result, observed performance gains primarily reflect improvements in layout understanding, structured extraction, and multimodal reasoning, and are influenced by the underlying capabilities of the base model.

Nevertheless, this choice aligns with current trends in document understanding, where end-to-end multimodal models are increasingly favored for complex, structured documents such as medical records.

Third, in regard to speed, deployability could be largely enhanced. While AWQ quantization helped compress the model in an efficient way, we acknowledge that our doctag tagging is computationally expensive due to the amount of tagging required. However, speed and efficiency could be easily enhanced by shortening the tag names such as <table> tag becoming <t>, <text>becoming <t>, class=“title” becoming cl=“t,” and similar variations. This would drastically reduce the amount of tokens required and speed up the model's inference time.

Finally, real-world validation relied on a limited set of publicly available document images that broadly matched the document categories used during synthetic data generation. To partially mitigate this limitation, we performed bootstrap resampling analysis which confirmed that the observed performance gaps between the fine-tuned model and all baselines are consistent and nonoverlapping across CIs, supporting the reliability of our conclusions within the available data. Although the sample size and sourcing reflect the practical difficulty of obtaining real clinical data, the evaluation shows clear cross-domain generalization and confirms that models trained exclusively on synthetic data can perform effectively on real-world medical documents.

Taken together, these considerations highlight opportunities for incremental refinement. In practice, insights from real-world deployment, such as institution-specific document layouts or information extraction requirement schemas, can be fed back into template design, placeholder definitions, and document class selection. This iterative process allows MedDocSynth to be adapted to specific institutional contexts while preserving its core objective of enabling privacy-preserving training for structured OCR and document-understanding systems.

### Conclusion

We presented a privacy-conscious, template-driven pipeline for generating realistic Korean medical documents with AI-filled content and structured XML annotations, addressing a key gap for clinical OCR. The resulting dataset spans diverse document classes and preserves domain-specific language and layout complexity, while lexical diversity analyses confirm rich free-text variability alongside expected regularity in structured fields. Together, these elements provide a practical foundation for training and evaluating OCR systems in hospital settings, enabling on-premise development without exposing patient data and offering a reusable framework that can be extended across institutions and tasks. Clinical deployment, including workflow integration and prospective evaluation, remains an important next step and a clear direction for future work building on this pipeline.

## Supplementary material

10.2196/91313 Multimedia Appendix 1 Supplemental tables S1-S6.

10.2196/91313 Multimedia Appendix 2 Examples of the data generated by our pipeline. Images from left to right: templates with placeholder annotations, generated documents with synthetic data, and XML-format annotations of the generated documents.

## Abbreviations

AWQ: aware weight quantization
CER: character error rate
COPE: Committee on Publication Ethics
DC: DocCreator
DS: DocSynth
EMR: electronic medical record
HIRA: Health Insurance Review and Assessment Service
JMIR: Journal of Medical Internet Research
MTLD: measure of textual lexical diversity
OCR: optical character recognition
ST: SynthText
STi: SynthTiger
TEDS: tree-edit-distance similarity
TR: TextRenderer
TRDG: TextRecognitionDataGenerator
TSTR: train-in-synthetic, test-on-real
TTR: type-token ratio
VLM: visual language model
WER: word error rate
