# Supplementary material for: Synthetic Medical Document Generation Pipeline (MedDocSynth) for Optical Character Recognition: Development and Validation Study on Korean Clinical Documents
Source: JMIR Med Inform. 2026 Oct 21;14:e91313. doi: 10.2196/91313 (PMC13686241; doi:10.2196/91313)
Supplement: Supplementary file 1 [file medinform-v14-e91313-s001.docx]

**Table S1.** Definitions and examples of XML tags and attributes used in the structured document representation.

| **Tag / Attribute** | **Description** | **Example** |
| --- | --- | --- |
| <page> | Root element that contains the entire document | (no attributes) |
| <text> | Text block used for titles or free-text answers | <text class="title"></text> |
| <table> | Container for tabular content | Contains <row> elements |
| <row> | A row inside a table | Contains <hcel> or <ncel> |
| <hcel> | Header cell used for labels or prompts | <hcel class="title"></hcel> |
| <ncel> | Normal cell containing answer values | <ncel class="answer" datatype="int">24</ncel> |
| **Attributes** |  |  |
| class | Specifies semantic role: either a title/label or an answer/value | class="title", class="answer" |
| datatype | Defines the type of data stored inside a cell or text block | int, date, string, code, gender, name |
| span | Column span that merges a cell across multiple columns | <ncel span="2" /> |

**Table S2.** Definitions and examples of supported placeholders in the synthetic document generation pipeline. the upper placeholders belong to clinical information related datatypes such as name, date, time, phone number, gender, address, code, amount, unit. The lower part describers other datatypes such as other strings, other integers, other float, arrow, symbol, range, combined fields and select.

| **Placeholder** | **Description** | **Example** | |
| --- | --- | --- | --- |
| Name | Full name of a person | Jane Doe | |
| Date | Date in numeric or natural format | 2025-05-09, May 9, 2025 | |
| Time | Time of day | 14:30 |  |
| Phone Number | Telephone number | +82-010-1234-5678 |  |
| Gender | Gender character or label | M, F, , |  |
| Address | Full postal address | 123 Main St, Seoul |  |
| Code | Alphanumeric or medical code | D1003, CURB-65 |  |
| Amount | Number with a unit or counter | 500mg, Hb6.7, 7 |  |
| Unit | Unit of measurement | mg, KWh |  |
| Other String | General free-text string | Hello, world! |  |
| Other Int | Integer number | 42 |  |
| Other Float | Floating-point number | 3.14 |  |
| Arrow Symbol | Directional symbol  Single-character symbol | −+,→-, @, < |  |
| Range | Numeric range | 1--7, 7--35 |  |
| Combined | Combination of multiple datatypes (shown in parentheses) | combined(int/gender) 58/M | → |
| Select | Single or multiple choice selection | □ Option A □ Option B | |

**Table S3.** Test set statistics by document category. Language composition is reported as percentages of total tokens (character-level), and structural statistics are derived from XML annotations.

Category	#Docs	Words	Language (%)	Tables	Docs w/ Tbl. (%)	Fields

|  |  |  | KO | EN | Other |  |  | (T/A) |
| --- | --- | --- | --- | --- | --- | --- | --- | --- |
| Surgical record | 4 | 2,592 | 28.2 | 56.7 | 15.1 | 2 | 25.0 | 96/94 |
| Pulmonary finding record | 1 | 560 | 74.3 | 3.2 | 22.5 | 2 | 100.0 | 24/15 |
| Physicians orders | 2 | 1,133 | 59.9 | 4.7 | 35.4 | 2 | 100.0 | 86/62 |
| Pathology results | 7 | 5,751 | 12.5 | 66.2 | 21.3 | 4 | 28.6 | 105/249 |
| Outpatient progress notes | 3 | 2,213 | 14.6 | 64.2 | 21.3 | 1 | 33.3 | 63/115 |
| Medical imaging | 9 | 6,120 | 20.6 | 62.5 | 17.0 | 2 | 22.2 | 151/190 |
| Medication records | 2 | 482 | 46.9 | 13.5 | 39.6 | 2 | 100.0 | 21/73 |
| Inpatient assessment | 6 | 3,724 | 34.7 | 45.6 | 19.7 | 1 | 16.7 | 132/162 |
| Emergency record | 8 | 3,938 | 33.0 | 45.6 | 21.5 | 5 | 37.5 | 168/199 |
| Diagnostic test results | 5 | 3,376 | 21.4 | 53.8 | 24.7 | 3 | 60.0 | 111/127 |
| Consultation records | 3 | 2,163 | 25.8 | 55.0 | 19.2 | 0 | 0.0 | 54/80 |

**Table S4.** Top bigrams and trigrams by datatype. Free-text entries show broad n-gram variety but also a small number of moderately frequent, clinically relevant phrases (e.g., “일 회” ― 78 occurrences out of 4,930), which is expected and mirrors real-world clinical usage; these repeated patterns contribute to high MTLD without indicating excessive templating. In contrast, structured fields such as addresses produce template-like geographic sequences (e.g., repeated “강남구” tokens), reflecting both narrow semantic roles and a dataset-specific geographic bias.

| **Datatype** | **Top Bigrams (2-grams)** | **Top Trigrams (3-grams)** |
| --- | --- | --- |
| String (4930 samples) | ‘일 회’ (78)  ‘하루 회’ (65)  ‘q h’ (64) | ‘mg 필요 시’ (33)  ‘U A EDTA’ (30)  ‘mg 하루 회’ (30) |
|  | ‘ibuprofen mg’ (63) | ‘매일 저녁 시’ (30) |
|  | ‘진행 중’ (59) | ‘일 회 식전’ (30) |
| Address (80 samples) | ‘서울특별시 강남구’ (21)  ‘강남구 테헤란로’ (19)  ‘동 호’ (13) | ‘서울 강남구 테헤란로’ (10)  ‘서울특별시 강남구 테헤란로’ (9)  ‘서울특별시 강남구 삼성로’ (7) |
|  | ‘서울 강남구’ (11) | ‘서울시 송파구 잠실동’ (6) |
|  | ‘부산 해운대구’ (9) | ‘부산광역시 해운대구 중동’ (3) |
| Combined name + string (10 samples) | ‘피부과 전문의’ (3)  ‘이영희 피부과’ (2)  ‘심리학과 교수’ (2) | ‘이영희 피부과 전문의’ (2)  ‘이영희 소아과 의사’ (2)  ‘이민수 심리학과 교수’ (1) |
|  | ‘이영희 소아과’ (2) | ‘이민재 심리학과 교수’ (1) |
|  | ‘소아과 의사’ (2) | ‘이민지 심리학 연구원’ (1) |

**Table S5.** Per-datatype F1-scores across all datatypes extracted from XML-labeled fields. Datatypes are grouped vertically for readability. Macro-F1 and Weighted-F1 are computed over the full datatype set. Best value per datatype in **bold**.

| **Model** | **Name** | **Date** | **Phone Code Amount** | | | **Time** | **Gender** | **String** | **Address** |
| --- | --- | --- | --- | --- | --- | --- | --- | --- | --- |
|  |  |  | **Common Datatypes** | | |  |  |  |  |
| Qwen2.5-VL-Finetuned | **0.7531** | **0.8493** | **0.8333** | **0.8095** | **0.4516** | **0.6977** | 0.4444 | **0.7582** | **0.8000** |
| Qwen2.5-VL-Finetuned-AWQ | 0.6753 | 0.7107 | 0.5455 | 0.7532 | 0.5000 | 0.6316 | **0.6667** | 0.6919 | 0.8000 |
| GPT-5-Mini | 0.7020 | 0.6885 | 0.0000 | 0.5532 | 0.0870 | 0.4242 | **0.7500** | 0.5729 | **1.0000** |
| GPT-5-Mini-3-Shot | 0.7049 | 0.8136 | 0.0000 | 0.7200 | 0.4000 | 0.4375 | 0.8889 | 0.6028 | 1.0000 |
| GPT-5-Mini-5-Shot | 0.7059 | 0.7978 | 0.0000 | 0.6849 | 0.1818 | 0.4375 | 0.8889 | 0.6004 | 1.0000 |
| Qwen2.5-VL | 0.5846 | 0.5238 | 0.0000 | 0.0000 | 0.0000 | 0.3030 | 0.6667 | 0.5196 | 0.5714 |
|  |  | **Remaining Datatypes and Overall Performance** | | | | |  |  |  |
| **Model** | **Char** | **Combined** | **Email** | **Float** | **Int** | **Range** | **Select** | **Symbol** | **Macro / Weighted F1** |
| Qwen2.5-VL-Finetuned | 0.0000 | 0.5556 | **0.6667** | 0.6154 | **0.8191** | **1.0000** | **0.9321** | 0.0000 | **0.6462** / **0.7792** |
| Qwen2.5-VL-Finetuned-AWQ | 0.0000 | 0.5263 | **0.6667** | 0.6667 | 0.7357 | 0.3243 | 0.6512 | 0.0000 | 0.5615 / 0.6780 |
| GPT-5-Mini | 0.0000 | 0.5926 | 0.0000 | **0.9231** | 0.7339 | 0.3030 | 0.2105 | 0.0000 | 0.4436 / 0.5644 |
| GPT-5-Mini-3-Shot | 0.0000 | 0.5672 | 0.0000 | 1.0000 | 0.8546 | 0.9600 | 0.3030 | 0.0000 | 0.5783 / 0.6323 |
| GPT-5-Mini-5-Shot | 0.0000 | 0.5294 | 0.0000 | 1.0000 | 0.7814 | 0.9600 | 0.0833 | 0.0000 | 0.5407 / 0.5975 |
| Qwen2.5-VL | 0.0000 | 0.3871 | 0.0000 | 0.0000 | 0.3874 | 0.0000 | 0.5926 | 0.0000 | 0.2668 / 0.4703 |

**Table S6.** AI prompts used in the MedDocSynth pipeline. The table presents the full prompt templates used for OCR-based XML annotation generation.

GPT-5-Mini

ROLE

You are a vision-language model designed to perform OCR and convert the extracted information into an XML annotation format. You must read the provided image carefully and output only the final XML without any explanation or commentary. The XML must follow the annotation rules described in the following paragraphs.

GENERAL ACCURACY

You must transcribe all text exactly as it appears, making no spelling mistakes and preserving all symbols. If the text includes the characters <, >, or , convert them to lt;, gt;, and amp;. Any content that is filled in or represents factual data should be treated as an answer. Always ensure symbols, punctuation, and spacing match the original unless they are aesthetic splits that should be merged logically.

TITLES AND ANSWERS

Separate content into titles and answers. A title is a field label or descriptor such as Name, Date of Birth, Patient ID, Department, or similar metadata. An answer is the filled-in value associated with that title. Name, date, time, age, gender, codes, phone numbers, addresses, numeric values, and any filled content must always be considered answers. When a title–answer structure exists, output a pair of <text class="title"> and <text class="answer"> tags. In paragraphs containing multiple similar data types such as dates, ensure all items in that paragraph use the same class consistently rather than mixing title and answer.

MERGING AND SPLITTING TEXT

If a sentence is broken into multiple visual lines but forms one continuous textual idea, merge it into a single <text> tag. If the content is clearly separated into multiple points, bullet lines, or indexed items, use separate <text> tags. When a single word is split across two lines for aesthetic reasons, write it as a normal continuous word.

TABLE DETECTION

A table is recognized only when it has at least two columns and when the grid organizes information in a way that still makes sense even if the decorative lines are removed. If text is arranged in columns purely for aesthetic layout and removing the columns would not change the meaning, then it is not a table. If there are lines clearly separating cells and the content is semantically tabular, then it should be treated as a table. Each independent table must be extracted separately. Tables use <table>, <row>, <hcel>, and <ncel>. Use <hcel> only for true header rows that do not follow a title–answer pattern. Otherwise use <ncel>. If a cell spans vertically across multiple rows, repeat its content in each row.

FREE TEXT OUTSIDE TABLES

Text that is not part of a table must be enclosed in <text> tags. These tags must have the class attribute set to either title or answer. If the text describes general narrative content such as impressions or notes, use class="answer" with datatype="string". Paragraphs should not be arbitrarily split unless there is a clear separation such as a new list item or an intentional break.

DATATYPES

Every <text class="answer"> tag must include a datatype attribute. Datatypes include string, date,

name, int, float, time, select, code, amount, arrow, combined, symbol, phone, unit, range, gender,

and address. When encountering a slash between two data components, such as 58/M, this must be

encoded as a combined datatype, for example datatype="combined(int/gender)". If a value includes a

measurement or a unit, use the amount datatype. Standalone symbols are assigned datatype="symbol".

Use the most appropriate datatype based on the semantic meaning of the content.

OUTPUT FORMAT

Your output must use the structure <?xml version="1.0" encoding="utf-8"?> followed by a single

<

page> element containing all <text> and <table> elements. No other wrapper tags are allowed. You

must output only the final XML and nothing else.

FINAL INSTRUCTION

Follow all rules strictly. Read the image thoroughly, extract all text and tables accurately,

classify each element correctly, apply datatypes consistently, detect tables precisely, merge or

split text where appropriate, encode special characters, and output only the final XML annotation.

SMALL EXAMPLE

The following is a short example of what your final output should look like when given an image

with a simple form.

?xml version="1.0" encoding="utf-8"?

>

<

page

>

<

<

text class="title">Patient Name</text

>

<

text class="answer" datatype="name">Kim Jiyoon</text

>

<

text class="title">Date of Birth</text

>

<

text class="answer" datatype="date">1975-06-21</text

>

<

table

>

<

row

>

<

hcel>Test</hcel

>

hcel>Result</hcel

>

<

/row

>

<

<

row

>

ncel>Blood Pressure</ncel

<

>

<

ncel datatype="string">120/80</ncel

>

/row

<

>

<

/table

>

<

/page

>

GPT-5-Mini-3-shot

NOTE: GPT-5-Mini-3-shot prompt is identical to GPT-5-Mini prompt until the FINAL INSTRUCTION

section. Afterwards it continues as follows:

EXAMPLES

The following are examples of what your final output should look like when given an image with a

simple form.

EXAMPLE 1:

<

?xml version="1.0" encoding="utf-8"?

>

<

page

>

<

text class="title">Patient Name</text

>

<

text class="answer" datatype="name">Kim Jiyoon</text

>

<

text class="title">Date of Birth</text

>

<

text class="answer" datatype="date">1975-06-21</text

>

<

table

>

<

row

>

<

hcel>Test</hcel

>

<

hcel>Result</hcel

>

<

/row

>

row

>

<

ncel>Blood Pressure</ncel

>

<

<

ncel datatype="string">120/80</ncel

>

<

/row

>

<

/table

>

<

/page

>

EXAMPLE 2:

<

?xml version='1.0' encoding='utf-8'?

>

page

>

<

text class="title"></text

>

<

<

text class="title">:</text

>

<

text class="answer" datatype="int">12347654</text

>

<

text class="title">:</text

>

<

text class="answer" datatype="name"></text

>

<

text class="title">:</text

>

text class="answer" datatype="combined(gender/int)">222333444</text

>

<

<

text class="title">FINDING:</text

>

<

text class="answer" datatype="string">(Follow up, S/P right anterior resection c

cholecystectomy on ––20150625)</text>

<

text class="answer" datatype="string"> Compared with previous outside CT on ––2002810,</text

>

<

text class="title"></text

>

<

text class="title">2018-04-04</text

>

<

/page

>

EXAMPLE 3:

>

?xml version='1.0' encoding='utf-8'?

<

page

>

<

<

table

>

<

row

>

>

<

hcel class="title"></hcel

<

ncel class="answer" datatype="int">30594938</ncel

>

/row

>

<

row

>

<

<

ncel class="title"></ncel

>

<

ncel class="answer" datatype="name"></ncel

>

<

/row

>

<

row

>

<

ncel class="title">/</ncel

>

<

ncel class="answer" datatype="combined" subtype="gender/int">/22</ncel

>

/row

>

<

row

>

<

<

ncel class="title"></ncel

>

<

ncel class="answer" datatype="string"></ncel

>

<

/row

>

<

/table

>

<

/page

>

\end{tcolorbox}

GPT-5-Mini-5-shot

NOTE: GPT-5-Mini-5-shot prompt is identical to GPT-5-Mini prompt until the FINAL INSTRUCTION

section. Afterwards it continues as follows:

EXAMPLES

The following are examples of what your final output should look like when given an image with a

simple form.

EXAMPLE 1:

<

?xml version="1.0" encoding="utf-8"?

>

<

page

>

<

text class="title">Patient Name</text

>

<

text class="answer" datatype="name">Kim Jiyoon</text

>

<

text class="title">Date of Birth</text

>

<

/row

>

<

/table

>

<

/page

>

EXAMPLE 5:

<

?xml version='1.0' encoding='UTF-8'?

>

<

page

>

<

text class="title"> </text

>

<

text class="title"></text

>

<

text class="answer" datatype="int">1212453</text

>

<

text class="title"></text

>

<

text class="answer" datatype="string"></text

>

<

text class="title"></text

>

<

text class="answer" datatype="date">2010-12-30</text

>

<

text class="title"></text

>

<

text class="answer" datatype="string">Histopath Exam</text

>

<

text class="title">GROSS DESCRIPTION</text

>

<

text class="answer" datatype="string">The specimen is submitted in five parts.

<

/page

>
